# Extended Use of the Wearable Cardioverter-Defibrillator: Which Patients Are Most Likely to Benefit?

**DOI:** 10.1155/2018/7373610

**Published:** 2018-11-29

**Authors:** Boldizsar Kovacs, Sven Reek, Nazmi Krasniqi, Urs Eriksson, Firat Duru

**Affiliations:** ^1^Arrhythmias and Electrophysiology Unit, Department of Cardiology, University Heart Center Zurich, University Hospital Zurich, Rämistrasse 100, 8091 Zurich, Switzerland; ^2^Department of Cardiology, Regional Hospital Wetzikon, Spitalstrasse 66, 8620 Wetzikon, Switzerland; ^3^Rain 34, 5000 Aarau, Switzerland

## Abstract

**Background:**

Wearable cardioverter-defibrillators (WCD, LifeVest, ZOLL) can protect from sudden cardiac death bridging a vulnerable period until a decision on implantable cardioverter-defibrillator (ICD) implantation can be reached. WCD is commonly used for 3 months or less. It is unknown, which patients use WCD longer and which patients are most likely to benefit from it.

**Hypothesis:**

Extended use of WCD is reasonable in selected cases based on underlying heart disease and overall patient risk profile.

**Methods:**

We conducted a systematic and comprehensive research of all published clinical studies on PubMed reporting on the use of the WCD. Only original articles reporting on wear times and time to appropriate shocks were included in our analysis.

**Results:**

The search resulted in 127 publications. 14 parameters were reported necessary for inclusion in our analysis. Median wear times ranged from 16 to 394 days. The median wear time was especially long for patients suffering from nonischemic cardiomyopathy (NICM) (range: 50–71 days) and specifically peripartum cardiomyopathy (PPCM) (120 days) and for heart transplant candidates. There was a large variation of appropriate shocks according to indication for WCD use. In contrast to NICM in general, the number of appropriate shocks was particularly high in patients with PPCM (0 in 254 patients and 5 in 49 patients, respectively). The median and maximal time periods to the first appropriate shock were longest in patients with PPCM (median time to the first appropriate shock: 68 days).

**Conclusions:**

Prolonged use of WCD is not uncommon in available literature. Patients suffering from NICM and specifically PPCM seem most likely to have longer therapy duration with WCD with success. Careful patient selection for prolonged use may decrease the need for ICD implantation in the future; however, prospective data are needed to confirm this hypothesis.

## 1. Introduction

The implantable cardioverter-defibrillator has firmly established itself as a treatment option for patients at high risk for life-threatening ventricular tachyarrhythmias. In fact, several large randomized-controlled studies have supported its efficacy in preventing sudden cardiac death (SCD) and in reduction of mortality in patients with ischemic cardiomyopathy (ICM) and an ejection fraction (EF) ≤35% at least 6 weeks after the ischemic event or nonischemic cardiomyopathy (NICM) and an EF ≤35% after at least 3 months of optimal medical therapy (OMT) in primary prophylaxis [1–5]. The place for ICDs in secondary prophylaxis is clear, and it is indicated when a hemodynamically relevant tachycardia is diagnosed without a reversible cause or within 48 hours of an ischemic event [6–8]. Several controversies remain, however, regarding the optimal use of ICDs in primary prevention. In particular, questions of risk stratification, including the optimal time point for implantation, are unresolved yet. Moreover, there are clinical scenarios where the use of an implantable cardioverter-defibrillator (ICD) is not possible.

**Figure 1:**
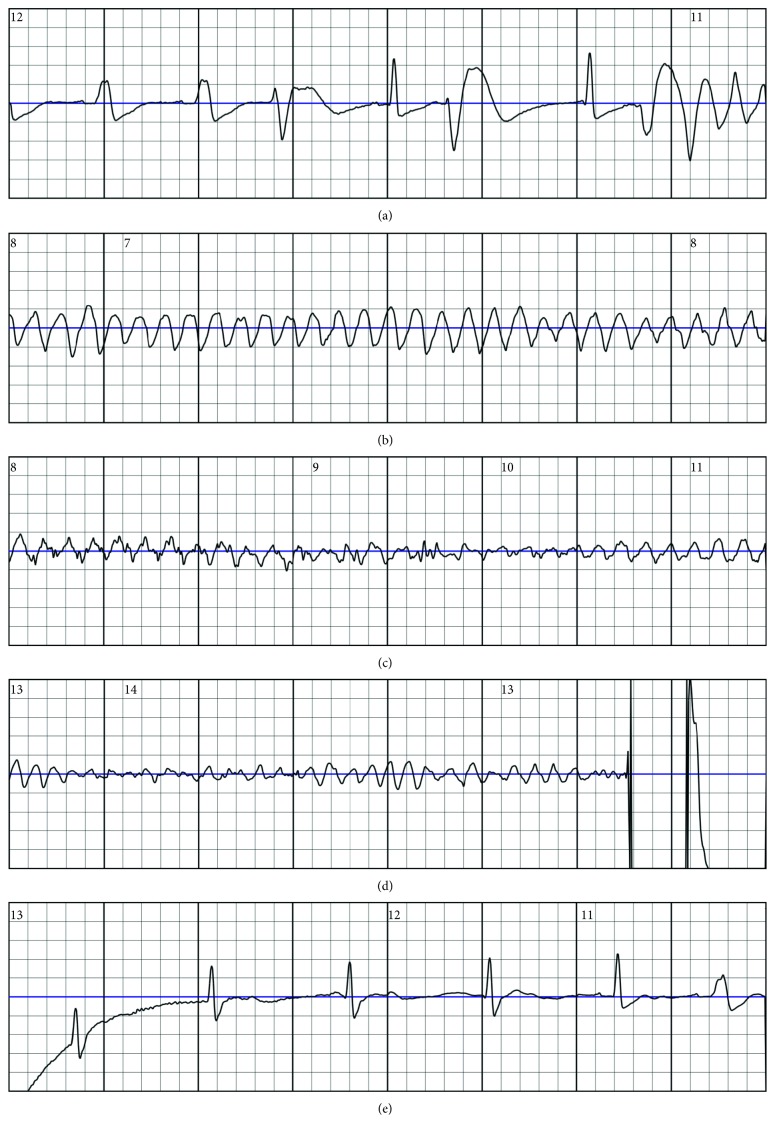
Ventricular flutter and fibrillation detected and appropriately terminated by a WCD in a patient.

The wearable cardioverter-defibrillator is a noninvasive device used temporarily for the prevention of SCD in presumed high-risk patients, which do not meet ICD implantation criteria on the basis of current guidelines. So far, only one wearable cardioverter-defibrillator (WCD) is approved for clinical use (LifeVest, ZOLL, Pittsburgh, Pennsylvania, USA). The device is a wearable vest with built-in electrodes for rhythm sensing, and pads in case a shock must be delivered [9, 10]. The WCD can detect ventricular tachycardia and ventricular fibrillation using an algorithm based on programmable heart rate cut-off values and ECG morphology analysis. Arrhythmia detection initiates a series of warnings before treatment ensues (Figure 1). Current recommendations, however, do not specify on the suggested WCD use duration except for ICM (40 days) [5]. On the contrary, WCD use is only a temporary protection against SCD. Prolonged use is defined as longer than the current recommended waiting period before implantation of an ICD, which is 3 months in the case of NICM and 40 days after the index event for ICM [11].

The aim of this study was to provide a systematic analysis of the published data on WCD-recorded appropriate shocks in different patient cohorts for extended time periods. The focus of our investigation was device wear length and time to the first appropriate shock. Our goal was to provide an overview of current data on prolonged WCD use in available literature and point out specific populations which seem to benefit most from it.

## 2. Methods

Studies were identified searching PubMed from the start of the database until the end of 2017 using the term “wearable cardioverter defibrillator” or “wearable cardioverter-defibrillator” and “wcd.” Only English-written articles were considered. Abstracts of all search results were screened and only original clinical studies, excluding case reports and small case series with *n* < 10 patients, were analyzed. All original articles examining the clinical use of WCD were screened for baseline characteristics, number of appropriate shocks, available median and maximal WCD wear times, for the median time to the first appropriate shock, and maximal time to the first appropriate shock. Our objective was to describe the prevalence of prolonged WCD use and the possible benefit of prolonged use. Prolonged use was defined based on current guidelines as longer than the recommended waiting period to ICD implantation, if indicated: 3 months in case of NICM and 40 days in case of ICM. Since no randomized clinical trials are available on this subject, only studies reporting either maximal wear time or median and maximal time to the first appropriate shock were included in our study, thus providing a summary of prolonged use of WCD and its potential benefit for specific populations.

## 3. Results

**Figure 2:**
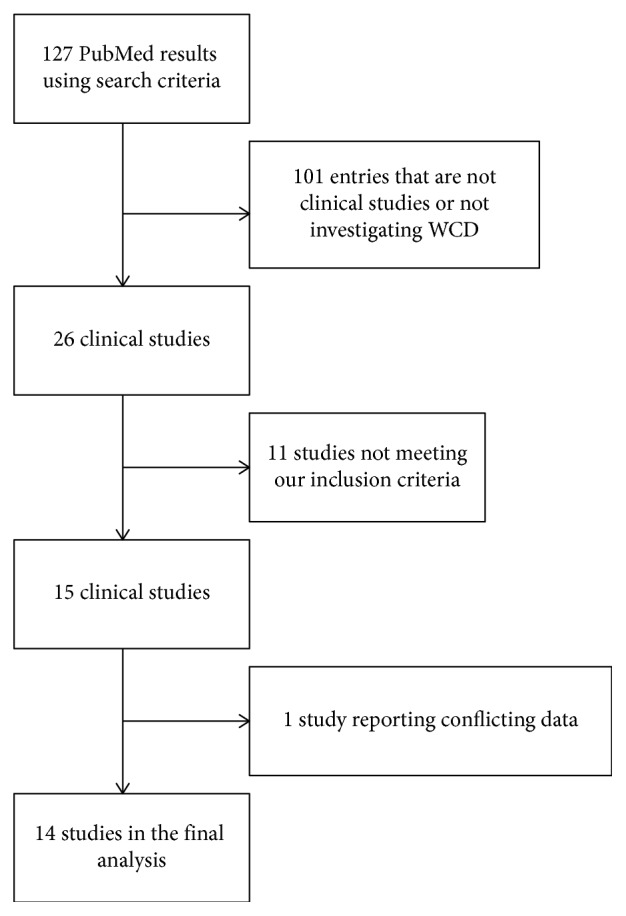
Study selection flow chart. WCD = wearable cardioverter-defibrillator.

The PubMed search resulted in a total of 127 publications. 101 were either not clinical studies or did not report on WCD use. The remaining 26 articles were screened, and 15 publications met the inclusion criteria. One study was excluded because of conflicting numbers reported in the dataset (the authors could not be reached for clarification) [12]. Study selection is shown in a flow chart in Figure 2.

The investigated 14 clinical studies were published between 2010 and 2017 (Table 1). 12 studies were retrospective based on registries, and data were collected by the manufacturer of the WCD device [13–24]. One study was a prospective observational investigation [25], and one study prescribed the WCD according to a prespecified algorithm and prospectively followed the study population [26]. Of the retrospective studies, one was labelled a review [24], but also reported on WCD experiences in Germany and fulfilled inclusion criteria. The main inclusion criterion in all studies was obviously WCD use, although the indications for use were variable. Seven studies included patients with any underlying heart disease as indication for WCD use, although one only included young patients, one only included patients of hemodialysis, and one examined patients with device infection only. The remaining five studies included patients with peripartum cardiomyopathy (PPCM), heart failure in general, and patients with ICM or NICM (Table 1).

The size of study populations ranged from 49 to 8453 patients in the investigated studies, and a total of 22908 patients were primarily reviewed for this analysis. All studies reported the median wear time (in 3 studies mean wear time [16, 21, 24]) ranging from 16 to 394 days and numbers of appropriate shocks (ranging from 0 to 309). Ten studies were reported on maximal wear time. 4 studies reported both median and maximal time to the first appropriate shock.

The prevalence of prolonged use was not specifically reported in either of the studies, except for the study of Lamichhane et al. which specifically only included long-term WCD users [18]. However, since three studies reported median wear times over 90 days and further six reported median wear times over 40 days, depending on the underlying condition prolonged use of WCD was highly prevalent. The shortest and longest median and maximal wear times and the shortest and longest maximal time to the first shock are summarized in Table 2.

The number of appropriate shocks per total study population varied greatly among the different indications. The highest rate observed was in patients on hemodialysis (136 appropriate shocks in 75 patients) [21]. A high rate was also observed in the study investigating patients with PPCM (5 appropriate shocks in 49 patients) [16]. No appropriate shocks were reported in one cohort with NICM, the only underage cohort (≤18 years), and in a mixed cohort of patients with heart failure [14, 19, 23]. Further study characteristics are illustrated in Table 1.

## 4. Discussion

We here provide the first systematic review of published data on prolonged WCD use in clinical practice and patients most likely to benefit from it.

### 4.1. Ischemic Cardiomyopathy

From the published data, it is evident that prolonged use of WCD was rarely reported in patients with ICM and ejection fraction ≤35%. This is not surprising, considering current guidelines giving an IA indication for ICD implantation 3 months after an MI or PCI/CABG with persistent symptomatic systolic heart failure based on the SCD-HeFT and MADIT-II trials [1, 2, 11]. Of the reviewed studies investigating WCD use in patients suffering from ICM, the majority report short median wear times and short times to the first appropriate shock. Epstein et al. reported a median time to the first appropriate shock of just 9 days after the start of the WCD use, which lies within the 40 days required by current guidelines before ICD implantation [22].

Singh et al. however report that only half of all shocks were in the first 40 days after an index MI suggesting that several cases of malignant arrhythmia occur after the initial waiting period. This difference to Epstein's results cannot clearly be explained, since EF and medication (including antiarrhythmic drugs) were not universally reported. Furthermore, the event rates differed by a factor of 2 between the two studies. Similar to what Singh et al. reported, appropriate shocks reported after an ischemic event occurred within 40 days in a European cohort investigated by Kondo et al. (not fulfilling inclusion criteria for primary analysis of this study) [27].

Lamichhane et al., on the contrary, specifically investigated the use of WCD beyond three months. In their study population, 35% had ICM, and the main reason for prolonged use was ongoing evaluation for ICD implantation. Only 6 patients received appropriate shocks in this cohort, and the authors, however, did not report the time of the events/shocks [18]. In light of this data, patients suffering from ICM seldom benefited from prolonged use of WCD. On the contrary, WCD possibly has benefits in the first 40 days after MI where the risk of SCD is reported to be up to 5% [28]. The recently published VEST [29]was the first randomized clinical trial assessing this question. They found no benefit in the prevention of SCD with the use of WCD in addition to guideline-directed therapy in the early stages after myocardial infarction, although therapy adherence was low. This result underscores the problem of patient selection and ensuring compliance and adherence to therapy.

### 4.2. Nonischemic Cardiomyopathy

The reported wear time for NICM was generally longer compared to ICM in the reviewed studies [13, 15, 19, 25, 30]. A direct comparison of the ICM and NICM population was subject only in the study of Singh et al. They noted that, within their total population, the wear time was significantly longer for NICM patients compared to ICM patients. The authors, however, did not observe shocks in this cohort. Chung et al. also did not report any shocks in the NICM subpopulation [15]. Wäßnig et al. found the longest wear times in the NICM subgroup but also low rates of appropriate shock. The WEARIT-II study reported 927 patients with NICM and WCD use. They report 1% arrhythmic events in this subpopulation, which was similar to Singh et al. and Wäßnig et al.'s data, and significantly less than for the ICM patient subpopulation.

In light of the DANISH study [31], the question arises whether a prolonged waiting/risk stratification period and establishment of OMT is warranted in this subgroup, before ICD implantation is considered. The prolonged study specifically examined patients with HFrEF after 3 months of WCD use with the goal of preventing unnecessary ICD implantations. An extended therapy was used in patients who did not yet have OMT or showed an improvement of EF since previous visit but still had an EF ≤35%. At the end of the follow-up (median 9 months), 33% of patients showed an improvement of EF to >35% [32]. They observed improvement more often in the NICM patients than ICM patients.

A further analysis from the WEARIT-II registry investigated patients using the WCD beyond 90 days and found a higher rate of extended use in NICM patients and furthermore discovered a further improvement in EF and thus obviating the need for an ICD in one-third of their patient cohort [33]. So far, the optimal timing of ICD implantation in NICM patients is still under debate. Whether prolonged WCD use in NICM patients is an option to prevent unnecessary ICD implantations, while minimizing the risk of sudden cardiac death remains an open question [34–36]. Hopefully, the currently recruiting HF-Opt trial (NCT03016754) examining EF improvement beyond 90 days of WCD use will hopefully provide some additional information on this subject.

Another possible indication for WCD use is PPCM, which however may have been reported in previous cohort investigating NICM as well. Duncker et al. provided the study examining only patients with PPCM [16]. They report a high prevalence of appropriate shocks. In contrast, in a study, Saltzberg et al. (not meeting inclusion criteria for primary analysis) compared patients with PPCM to NICM of other aetiology. The 107 patients included in their analysis wore the WCD 75 ± 81 days and had no events requiring intervention from the device [37]. It is noteworthy that this study was a retrospective analysis with patient inclusion based on the International Classification of Diseases coding and not according to current ESC criteria which may explain the much lower event rate compared to what Duncker et al. reported from their prospective study. Although the few other patient characteristics reported by all of the aforementioned three studies were similar (age, baseline EF, and parity), a more precise comparison is not feasible due to pronounced differences in methodology, reported variables, and outcome measures. Of note, another study reporting on cases of PPCM also reported on a high rate of appropriate shocks during prolonged wear time [38].

Current ESC guidelines recommend ICD implantation following standard guidelines for NICM, but also refer to WCD treatment (recommendation level class IIb). This is particularly noteworthy since EF recovery can be expected in a high rate of PPCM patients [11].

### 4.3. WCD in Patients with Device Infection

Data regarding WCD use and device removal were available in 6 studies, while Tanawuttiwat et al. only examined this cohort [13, 15, 20, 21, 24, 25]. The largest number of patients examined was provided by Wäßnig et al. [13]. Compared to the study of Ellenbogen et al. not meeting inclusion criteria for primary analysis, the median wear time was comparable but longer than what Tanawuttiwat et al. reported. Ellenbogen et al. demonstrated that 22% of their patients wore the WCD for over 3 months without reporting on the indication for this [39]. For obvious reasons, a prolonged wear time is only necessary in selected cases since the indication for ICD implantation has previously been made. Still an extended use is clearly indicated until the infection abates and surgery is again possible.

### 4.4. Heart Transplantation

Bridging the time to heart transplantation is another possible indication for WCD use [23, 24]. Although the reported number of appropriate shocks was low, since the perioperative risk of ICD implantation is especially high, these patients could especially benefit from extended WCD use. The ESC guidelines recommend an ICD implantation for all patients listed for transplant in a New York Heart Association class IV, whereas WCD use as a bridge to transplant is an alternative (recommendation level class IIa and IIb, respectively). In our opinion, extended wear for secondary prophylaxis of SCD is also an option in these patients.

### 4.5. Renal Failure

There are other, less frequent indications for WCD use. Wan et al. investigated patients on hemodialysis [21]. This patient group is not included in most pivotal ICD trials. They found that the main reason for WCD use was active infection and contraindication for ICD implant in this cohort, but they also pointed out that majority of patients used the device for an extended period of time until EF improvement or ICD implantation. Patients with end-stage renal disease represent a high-risk population for ICD implantation explaining the impressively high prevalence of arrhythmic events during the study period. Nevertheless, the same guidelines apply for ICD implantation in these patients as for the general population. Thus, prolonged WCD use was only considered in few cases in available literature.

### 4.6. Myocarditis

Myocarditis was infrequent in the reviewed studies. Only two studies were reported on wear time [13, 25]. The current ESC guidelines give a weak recommendation for the use of WCD in these patients, similar to the PPCM population. Given the excellent long-term outcome of patients with myocarditis who recover from the impaired cardiac function, prolonged WCD use may be reasonable in this population.

### 4.7. Inherited Channelopathies

Few patients with channelopathies or congenital heart diseases were included in the assessed studies [15, 24, 25]. It seems prudent to thoroughly evaluate these patients before ICD implantation, while protecting them from SCD if necessary. To facilitate a safe risk stratification, WCD might be an option. Depending on the phenotype of underlying disease, this stratification period can be rather long depending on clinical judgment.

### 4.8. Children and Adolescent

Another population who may benefit from an extended use of WCD is children and younger adults. While they may have the highest lifetime benefit of an ICD implantation if indication is made correctly, they are also at higher risk of ICD complications such as multiple battery changes and thus increased risk of infection. Collins et al. examined a population ≤21 years of age with a WCD use for any indication. The main causes of increased arrhythmogenic risk were cardiomyopathy, primary arrhythmia (without specification), and congenital heart disease especially in the ≤18 years of age cohort. Since at the end of their study only 32% of patients had an ICD implanted, prolonged WCD use might be justified [14].

### 4.9. Summary of Reported Prolonged WCD Use

Several patient populations were reported to use the WCD for an extended period. Some subgroups were more prevalent probably due to the (assumed) reversibility of their elevated arrhythmogenic risk. In general, patients not yet receiving an OMT deserve a chance to improve their ejection fraction while being protected from SCD events. Patients possibly benefiting prolonged use after review available literature may be patients with PPCM and young patients in order to avoid unnecessary ICD implantation. These patients may be at higher risk of dying from SCD than dying of other cause, yet ICD implantation should be carefully evaluated after establishing OMT.

Clinicians also have to take into account patients' choice to refuse ICD while accepting a longer WCD use. The prolonged use of WCD in patients with NICM seems less clear particularly due to low treatment rates reported. There are several disadvantages of a prolonged WCD use. Wear comfort is an obvious problem especially over longer periods. The absence of pacing modalities (for bradycardia, antitachycardia pacing, or postshock pacing) can be an issue and fail to prevent SCD due to asystole. Lastly, a cost-benefit analysis is necessary to justify a longer WCD use.

### 4.10. Limitations

Our analysis has several limitations. The heterogeneity of clinical studies, which resulted in missing data on the time of appropriate shocks, is a limitation of this study. 11 of the 14 studies reported the database kept by ZOLL. It is therefore possible that patients fulfilling inclusion criteria for more than one of the listed studies in Table 1 were reported more than once.

## 5. Conclusions

Extended use of WCD is commonly reported in reviewed literature, although majority of it is of descriptive nature. Patients most likely to wear the device longer than 3 months seem to be patients with NICM, specifically PPCM likely due to the lack of other significant comorbidities and the high rate of disease improvement beyond the first 3 months often obviating the need for ICD implantation. On the contrary, patients listed for heart transplantation could also benefit from prolonged therapy to avoid risks of the more invasive ICD implantation. Question remains, however, what the rate of appropriate shock is during this prolonged use, if a true benefit is present compared to ICD implantation. To assess this true benefit, prospective and randomized data are needed.

## Figures and Tables

**Table 1:** Studies reviewed.

Year	Author	Indication	*n*	Median wear time	Maximal wear time	Number of appropriate shocks	Median time to the first appropriate shock	Maximal time to the first appropriate shock
2010	Collins et al. [14]	Any (≤18 years of age)	81	29	531	0	No shocks	No shocks
		Any (18–21 years of age)	103	35	499	5	^*∗*^	^*∗*^
2010	Chung et al. [15]	Any	3569	36	1590	80	^*∗*^	^*∗*^
2010	Klein et al. [24]	Any	354	106^a^	>7 years	21	^*∗*^	^*∗*^
2010	Dillon et al. [17]	Any	2105	36	365	54	^*∗*^	^*∗*^
2012	Kao et al. [23]	Heart failure^b^	82	64	277	0	No shocks	No shocks
2013	Epstein et al. [22]	ICM	8453	57	^*∗*^	309	9	>9 months
2014	Wan et al. [21]	Hemodialysed patients^c^	75	62.9^a^	308	136	^*∗*^	^*∗*^
2014	Tanawuttiwat et al. [20]	Device infection	97	21^d^	^*∗*^	4	23	38
2015	Singh et al. [19]	ICM	271	53	^*∗*^	6	34	45
		NICM	254	71	^*∗*^	0	No shocks	No shocks
2016	Lamichhane et al. [18]	HFrEF^e^	220	394	2013	13	^*∗*^	^*∗*^
2016	Wäßnig et al. [13]	Any	6043	59	163	^*∗*^^f^	^*∗*^	^*∗*^
2017	Erath et al. [25]	Any	1102	54	166	8	^*∗*^	^*∗*^
2017	Sasaki et al. [26]	Any	50	16	171	6	12	30
2017	Duncker et al. [16]	PPCM	49	120^a^	^*∗*^	5	68	124^g^

^a^Reported as a mean; ^b^defined by own specific criteria; ^c^only patients with SCD events included; ^d^median calculated for only 80 study patients; ^e^wear time always >90 days; ^f^89% of treatments occurred in the first 90 days; ^g^calculated from the time of diagnosis and not from the beginning of device therapy; ^*∗*^not reported. Time is presented in days.

**Table 2:** Shortest and longest device parameters reported in the included studies.

	Median wear time	Maximal wear time	Median time to the first appropriate shock	Maximal time to the first appropriate shock
Shortest	16	163	9	30
Longest	394	>7 years	68	>9 months

## Data Availability

The data used to support the findings of this study are included within the article.
